# Long: Influence of water masses on the summer structure of the seabird community in the northeastern Chukchi Sea

**DOI:** 10.1371/journal.pone.0266182

**Published:** 2022-04-07

**Authors:** Adrian E. Gall, Alexander K. Prichard, Katherine J. Kuletz, Seth L. Danielson

**Affiliations:** 1 ABR, Inc.—Environmental Research & Services, Fairbanks, Alaska, United States of America; 2 Migratory Bird Management, U. S. Fish and Wildlife Service, Anchorage, Alaska, United States of America; 3 College of Oceanic and Atmospheric Sciences, University of Alaska Fairbanks, Fairbanks, Alaska, United States of America; Hawaii Pacific University, UNITED STATES

## Abstract

We used data collected during a variety of research cruises in the northeastern Chukchi Sea and contributed to the Distributed Biological Observatory to explore the influence of the seasonal change in water masses on the development of the seabird community during the summer. Surveys that included seabird observations and hydrographic sampling were conducted from Alaska’s northwestern coast to ~220 km offshore during 2008–2018. Species composition varied geographically, shifting from a nearshore community that included short-tailed shearwaters, loons, and seaducks to an offshore community dominated by crested auklets. Crested auklets were remarkably consistent in their occupation of Hanna Shoal among years and remained in the area throughout the summer. Short-tailed shearwaters exhibited the greatest seasonal and interannual variation in abundance and distribution of the 35 species recorded. They were concentrated south of 71°N and within 50 km of shore in August and tended to spread throughout the region in September. Surface-feeding species like gulls, fulmars, and phalaropes were 1–2 orders of magnitude less abundant and had wider distributions than birds that feed by diving. Including information about hydrography improved the fit of models of seabird density. Seabirds, especially those that breed in the Bering Sea, generally were more abundant in areas dominated by moderate-salinity Bering Sea Water than nearshore in low-salinity Alaska Coastal Water. The distribution of seabirds across the northeastern Chukchi Sea reflected the heterogeneity of oceanic habitats and prey availability over the shallow shelf. Our results will inform efforts to develop ecosystem models that incorporate oceanographic conditions to predict ongoing consequences of climate change.

## Introduction

Seabird distribution across a seascape can reflect oceanographic conditions at lower trophic levels, serving as visible indicators of marine ecosystems that are otherwise obscured under water [1–3]. The northeastern Chukchi Sea is being altered by fundamental changes in the regional climate that are restructuring the marine food web by creating an environment that is warmer, fresher, and more ice-free than in the previous three decades [4]. The rate of warming has been accelerating in recent years and decades [5]. These changes are affecting processes that influence the distribution, life history, and interactions of biological communities [6–9]. Declining seasonal ice cover also is increasing access to the Chukchi Sea, providing new opportunities for human activities such as recreational boating, commercial shipping and fishing, and oil and gas exploration. The seabird community offers benchmarks for evaluating both the short-term effects of catastrophic events such as oil spills and the long-term responses to climate change.

The eastern Chukchi shelf sustains a diverse seabird community during the July–October open-water season [10–12]. A few species of piscivorous seabirds such as murres (*Uria* spp.), puffins (*Fratercula* spp.), and black-legged kittiwakes (*Rissa tridactyla*) nest in large colonies (~500,000 birds) at Cape Thompson and Cape Lisburne to take advantage of the fish available in nearshore waters [13–15]. Other species-groups such as jaegers (*Stercorarius* spp.), gulls (*Larus* spp.), and loons (*Gavia* spp.), nest on the tundra and forage in the marine environment during or after the breeding season [16]. In addition to breeding seabirds, non-breeding and post-breeding seabirds move into the northern Chukchi Sea as the ice recedes to feed on both fish and zooplankton [10–12]. This community of >40 species of seabirds depends on a variety of habitats created when warm water masses move northward from the Bering Sea [17] and interact with cold water masses of the northern shelf formed during winter [18,19] and modified by nearshore warming during spring and summer. Together, these physical processes form four major water masses that drive the environmental gradients of the Chukchi Sea.

**Fig 1:**
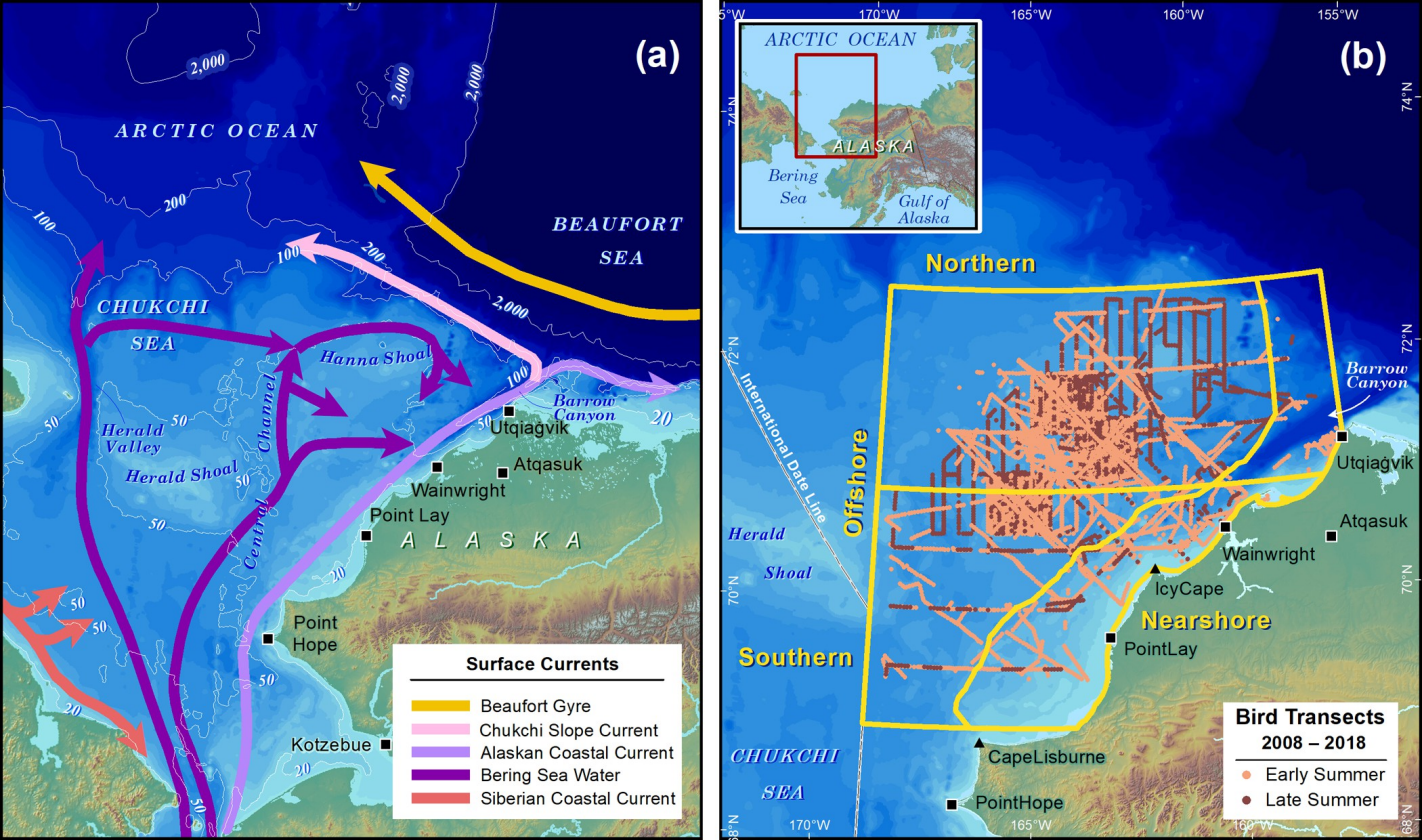
The Chukchi Sea, showing (a) current locations; and (b) geographic strata and survey effort. Bathymetry data are from the International Bathymetric Chart of the Arctic Ocean www.ibcao.org.

The four water masses within the study area in the summer differ in temperature, salinity, and stratification, which are key determinants of foraging habitat [20]. The Alaska Coastal Current (ACC) lies adjacent to the Alaska coastline and flows northward, carrying Alaskan Coastal Water (ACW), a warm (>7°C), low-salinity (<30.8) water-mass [5] that originates south of Bering Strait and is additionally supplied by fresh river outflows as it progresses northward. The currents farther offshore move Bering Sea Water (BSW; [17,21]), a moderately warm (0–7°C) and moderate-salinity (30.8–33.4) water mass [5], northward through the Central Channel and Herald Valley (Fig 1; [22]). One branch of the BSW pathway is an eastward flow south of Hanna Shoal [23,24]. BSW is often a mixture of Anadyr Water and Bering Shelf Water from south of Bering Strait; it has an elevated nutrient content and transports more and larger oceanic zooplankton than do the ACW flows [25,26]. Water masses are modified on the Chukchi shelf in the winter when ice formation produces cold (~- 2–0°C) and brine-enriched, Winter Water (WW). Ice melts and leaves cool (0–3°C), low-salinity (<30.8) Meltwater (MW) at the surface [5,27] that helps regulate the exchange of heat between the BSW and the pack ice [28]. These four water masses (ACW, BSW, WW, and MW) provide habitat for a seasonally diverse assemblage of seabirds. Dynamic fronts and flow instabilities occur at the boundaries between the Chukchi water masses [28], which can concentrate plankton and increase foraging opportunities for surface-feeding and near-surface-feeding seabirds.

Seabird prey communities associated with these water masses also differ substantially and likely contribute to determining the distribution and composition of seabird communities. Prey species associated with ACW include small neritic copepods and a variety of forage fishes that include 5 species of salmon (*Oncorhynchus* spp.), rainbow smelt (*Osmerus mordax*), Pacific sandlance (*Ammodytes hexapterus*), and Arctic cod (*Boreogadus saida*; [29,30]. Salmonids are found almost exclusively in the surface waters of the shelf [30,31], whereas other forage fish are found throughout the water column. The low temperatures of two-layered MW/WW near Hanna Shoal preclude the development of a diverse fish community [8,32]. In contrast, the pelagic community is characterized primarily by cold-tolerant Arctic cod and the seasonal development of a zooplankton community that includes the large arctic copepod *Calanus glacialis* [33]. BSW is intermediate in temperature and salinity between WW and ACW and transports energy-rich Pacific zooplankton prey, including *Neocalanus* copepods and euphausiids, into the study area [34].

The biological communities found on the broad shelf of the northeastern Chukchi Sea are structured by the northward flow of Pacific water and the seasonal advance and retreat of sea ice [35–38]. These simple food webs are now being disrupted by increases in advection through Bering Strait and changes in sea ice regimes [36]. The Distributed Biological Observatory was established in 2010 as a change detection array to develop consistent time series for exploring the ecological consequences of climate change [39–41]. One of the strengths of the framework is a holistic approach that seeks to link measurements of oceanography with data on species composition and distribution. We leveraged data collected on hydrography and seabirds in the northeastern Chukchi Sea to explore the influence of the seasonal change in water masses on the development of the seabird community during the open-water season.

We examined the distribution, abundance, and community composition of seabirds in the Chukchi Sea from Alaska’s northwestern coast to ~220 km offshore during 2008–2018. Herein we describe temporal and spatial changes in seabird species-composition along the nearshore–offshore oceanographic gradient and with respect to hydrographic conditions. By relating the temporal response of the seabird community to the intrusion and distribution of BSW, models that predict future oceanographic conditions may be applied to predict possible changes in the timing and composition of seabird communities as the Arctic continues to warm.

## Methods

### Study area

This study was conducted in the northeastern Chukchi Sea in an area bounded near the village of Point Lay in the south (69.922°N 162.578°W) and the Chukchi shelf break in the north (72.866°N 156.648°W), with data collection focused in an area extending from Alaska’s northwestern coastline westward to the U.S.–Russia maritime boundary (168.976°W, Fig 1). For comparisons of community composition, we divided the study area into four geographical/ecological strata to account for the effects of latitude, water masses, currents, and bathymetry on determining oceanic habitat [38]. First, we divided the area along the 40-m isobath running roughly parallel to shore. Although the exact location of the front between ACW and the offshore water-masses (BSW, MW, WW) may change within and among years [19], the 40-m isobath approximates the composite location of this front over time. Next, we divided the area by latitude along 71°N, an area of persistent eastward flow from the Central Channel to Barrow Canyon [22,24]. The resulting strata have distinct hydrographic characteristics, which we expected would influence and differentiate seabird communities within each one.

The Southern Offshore stratum has an area of 35,059 km^2^ and is characterized by northward flow of BSW through the Central Channel that then splits as it approaches Hanna Shoal, with some flow turning east toward the head of Barrow Canyon. The Southern Nearshore stratum has an area of 25,405 km^2^ and is influenced by northward coastal flows that carry predominantly ACW, although episodic flow reversals can transport slope waters southward from Barrow Canyon [42,43]. The Northern Offshore stratum has an area of 67,625 km^2^ and is influenced by the anticyclonic flow around Hanna Shoal [44] and resident MW/WW over the shoal that drains into Barrow Canyon from the shelf throughout the summer [27]. The Northern Nearshore stratum includes the head of Barrow Canyon and the eastern end of the Chukchi continental slope, an area of high biological productivity that supports feeding aggregations of seabirds and marine mammals [12].

### Data collection

Oceanographic data and data from systematic seabird surveys were pooled across various research programs conducted during 2008–2018 (Table 1). We surveyed a total of 35,680 km across years (11,893 3-km transects), with all surveys conducted from 13 August to 2 October. Seabird surveys followed protocols established and refined by the U. S. Fish and Wildlife Service [45,46] using vessels 35–128 m long and in waters at least 6 m deep. The closest approach to shore was 1.3 km and no permits were required to operate in Federal or State waters. A small number of transects extended into the Ledyard Bay Critical Habitat Unit, an area managed by the U. S. Fish and Wildlife Service (USFWS) for the protection of molting spectacled eiders. We engaged in an informal consultation with the USFWS to confirm best practices while operating in the bay; no special permits were required.

**Table 1:** Sampling effort by year, northeastern Chukchi Sea, 2008–2018.

Year	Date start	Date end	Number of 3-km transects	Area surveyed (km2)
2008	16-Aug-2008	28-Sep-2008	838	623
2009	13-Aug-2009	2-Oct-2009	1,484	1,172
2010	13-Aug-2010	2-Oct-2010	1,749	1,374
2011	13-Aug-2011	2-Oct-2011	1,633	1,188
2012	15-Aug-2012	1-Oct-2012	2,368	1,784
2013	13-Aug-2013	2-Oct-2013	1,329	989
2014	20-Aug-2014	23-Sep-2014	348	682
2015	13-Aug-2015	3-Sep-2015	1,014	823
2016	12-Sep-2016	13-Sep-2016	45	37
2017	13-Aug-2017	21-Sep-2017	979	739
2018	13-Aug-2018	25-Aug-2018	106	75
Total			11,893	9,485

We conducted seabird surveys as continuous sampling when the ship was moving along a straight-line course at a minimum speed of 9.3 km/h [45,47]. These survey lines subsequently were split into 3-km sampling units (transects) for analysis using GIS because seabird communities are considered spatially independent at scales ≥ 3 km [48–50].

We collected data 9–12 h/day during daylight hours, weather and ice conditions permitting. Surveys generally were stopped when sea state was Beaufort 6 (seas ~2–3 m) or higher. One observer stationed on the bridge of the ship recorded all birds seen within a radius of 300 m in a 90° arc from the bow to the beam on the port side of the ship (the count zone) and located and identified seabirds with 10× binoculars. For each bird or group of birds, we recorded species (or identity to lowest possible taxon); total number of individuals; distance from the centerline (in categories; 0–50 m, 51–100 m, 101–150 m, 151–200 m, 201–300 m); location (air, water, flotsam/jetsam, ice); and behavior (flying, sitting, swimming, feeding, comfort behavior, courtship behavior, other).

We counted all birds on the water within the count zone, taking care to avoid recounting the same individuals. For flying birds, however, we conducted scans ~1 time/min (the exact frequency varied with ship’s speed) and recorded an instantaneous count (“snapshot”) of all birds flying within the count zone. This snapshot method reduces the bias of overestimating the abundance of flying birds [45,47]. We counted only those flying birds that entered the count zone from the sides or front and did not count those that entered from behind the ship (i.e., an area that already had been surveyed) to avoid the possibility of counting ship-following birds. We recorded observations of all birds directly into a computer connected to a global positioning system (GPS) with TigerObserver software (TigerSoft, Las Vegas, NV) or DLog (Glenn Ford, Seattle, WA). These programs time-stamped and georeferenced every observation entered in real time and provided a trackline of sampling effort.

Hydrographic data came from stations spaced 25–50 km apart, depending on the cruise. Conductivity-temperature-depth (CTD) measurements were made with a Sea-bird (SBE) 911 or SBE 25 CTD sampling at 24 and 4 Hz, respectively, that was lowered through the water-column at a rate of ~0.5 m s^-1^ to within 5 m of the seafloor. We measured pressure, temperature (± 0.005°C), and conductivity (S/m) and then computed depth and salinity (±0.02). Data collected with the CTD were processed according to the manufacturer’s recommendations and screened for anomalous spikes, dropouts and density inversions. We averaged the station data to 1-decibar (~1-m) vertical profiles that were then used to calculate the summary values for temperature, salinity, and density gradient.

### Data analysis

We selected transects that were conducted within a study area covering 140,582 km^2^, during days of year 225–275 (13 August–2 October), and had associated oceanographic data collected in situ within 2 days and at stations within 20 km of the respective transect centroid. To explore the influence of seasonal changes in water masses on the distribution of seabirds, we divided the study period into two 25-day periods: early summer (13 August–6 September) and late summer (7 September–2 October). In August, waters are typically the warmest and most ice-free and in September, waters tend to cool as days get shorter.

We limited the analysis to species that forage in the marine environment, and specifically in the Chukchi Sea. These included Scolopacidae (phalaropes), Stercorariidae (jaegers), Alcidae (auks), Laridae (gulls, terns), Gaviidae (loons), Procellariidae (fulmars, shearwaters), and marine species of Anatidae (eiders, scoters, other seaducks) (Table 2). All data processing, analysis, and statistical tests were performed in program R version 4.0.3 [51], with significance of p<0.05. Means are presented ± standard error (SE). Maps were created using ArcGIS v. 10.8 and other results figures were produced using package ‘ggplot2’ in R [52].

**Table 2:** Species of seabirds recorded during ship-based surveys in the northeastern Chukchi Sea, 2008–2018.

Family	Scientific name	English name	Code	Total count	
				Early summer	Late summer
Sea ducks	*Somateria fischeri*	Spectacled Eider	SPEI	21	17
	*Somateria spectabilis*	King Eider	KIEI	3	45
	*Somateria mollissima*	Common Eider	COEI	59	51
	*Clangula hyemalis*	Long-tailed Duck	LTDU	137	300
Phalaropes	*Phalaropus lobatus*	Red-necked Phalarope	RNPH	655	591
	*Phalaropus fulicarius*	Red Phalarope	REPH	529	180
Jaegers	*Stercorarius pomarinus*	Pomarine Jaeger	POJA	105	22
	*Stercorarius parasiticus*	Parasitic Jaeger	PAJA	26	4
	*Stercorarius longicaudus*	Long-tailed Jaeger	LTJA	10	2
Alcids	*Alle alle*	Dovekie	DOVE	13	3
	*Uria aalge*	Common Murre	COMU	283	92
	*Uria lomvia*	Thick-billed Murre	TBMU	1,892	1,102
	*Cepphus grille*	Black Guillemot	BLGU	7	10
	*Brachyramphus brevirostris*	Kittlitz’s Murrelet	KIMU	95	79
	*Synthliboramphus antiquus*	Ancient Murrelet	ANMU	50	441
	*Aethia psittacula*	Parakeet Auklet	PAAU	81	89
	*Aethia pusilla*	Least Auklet	LEAU	2,735	2,184
	*Aethia cristatella*	Crested Auklet	CRAU	25,642	18,885
	*Fratercula corniculate*	Horned Puffin	HOPU	66	5
	*Fratercula cirrhata*	Tufted Puffin	TUPU	24	2
Gulls	*Rissa tridactyla*	Black-legged Kittiwake	BLKI	1,021	1,931
	*Pagophila eburnea*	Ivory Gull	IVGU	0	3
	*Xema sabini*	Sabine’s Gull	SAGU	167	12
	*Rhodostethia rosea*	Ross’s Gull	ROGU	0	314
	*Larus brachyrhynchus*	Short-billed Gull	SBGU	1	0
	*Larus argentatus*	Herring Gull	HEGU	9	26
	*Larus hyperboreus*	Glaucous Gull	GLGU	174	499
	*Sterna paradisaea*	Arctic Tern	ARTE	75	3
Loons	*Gavia stellata*	Red-throated Loon	RTLO	1	8
	*Gavia pacifica*	Pacific Loon	PALO	50	492
	*Gavia immer*	Common Loon	COLO	1	4
	*Gavia adamsii*	Yellow-billed Loon	YBLO	4	48
Procellariiids	*Fulmarus glacialis*	Northern Fulmar	NOFU	906	246
	*Ardenna tenuirostris*	Short-tailed Shearwater	STSH	17,282	11,171

#### Community analyses

We used descriptive statistics and multivariate analyses to explore spatial and temporal variation in the seabird community. We first calculated sample-based rarefaction curves to evaluate species richness between seasons. This approach accounts for variation in sampling effort by resampling 3-km segments without replacement to estimate the rate at which species are detected [53,54]. For individual observations not identified to species, we retained the higher-order taxon only if no individuals of that group were identified to species [55].

For multivariate community analyses, we included only bird observations that were identified to species. Species that occurred in < 5% of groups or had fewer than 10 records were excluded from the analysis; these were short-billed gulls, ivory gulls, common loons, dovekies, and red-throated loons. We grouped data by geographic stratum, season, and year for ordination using non-metric multidimensional scaling (nMDS) [56]. The log-transformed species densities were used to calculate a Bray-Curtis similarity matrix [57] and then mapped those distances in two-dimensional space. The stress coefficient of the ordinations was 0.118, indicating adequate fit to the data [58]. We examined the variation in species composition among geographic strata and seasons with permutational multi- variate analysis of variance (PERMANOVA), which partitions variation and requires no assumptions about the distribution or correlations among the variables [59]. Finally, we visualized species composition by geographical stratum and season. We did not include 2017 in the species composition summary figures because in late summer 2017, short-tailed shearwaters were remarkably more abundant (by 1–2 orders of magnitude) and widespread than in other years, swamping all other species. We used package ‘vegan’ v.2.5–7 [60] for community analyses and packages ‘vegan’ and ‘ggplot2’ [52] for visualizations.

#### Abundance and distribution

We assigned 3-km transects to cells in a hexagonal grid overlaid on the study area based on the location of the transect centroid. Each grid cell measured 30 km from vertex to vertex. Hexagons have lower sampling bias at edges than do rectangular cells [61]. For each cell, we calculated the density of seabirds for each season and year as the total of birds observed on those transects within the cell divided by the total area surveyed.

**Fig 2:**
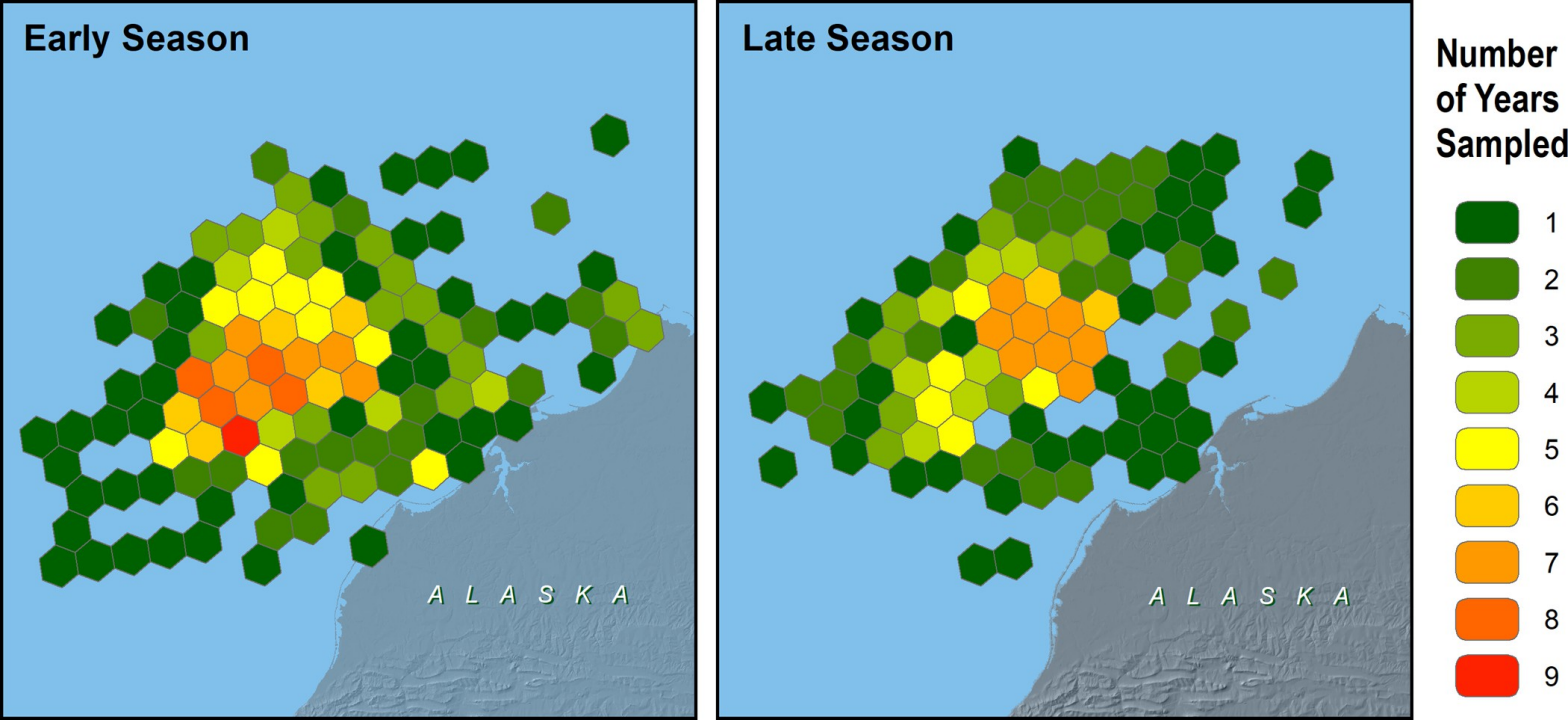
Seasonal and interannual differences in sampling effort in the northeastern Chukchi Sea, 2008–2018.

To avoid bias from over-inflated densities in hexagons with little surveyed area, we limited analysis of abundance and distribution to cells that had a minimum of 5 km^2^ of transect area sampled during a given season and year. There were 320 hexagons in early summer and 236 hexagons in late summer with adequate samples of transect data to include in density models. These hexagons were surveyed between 1 and 7 years each (Fig 2).

We selected 8 focal taxa for statistical analyses that together represent 95% of the seabird community: crested auklet (*Aethia cristatella*), least auklet (*A*. *pusilla*), phalaropes (*Phalaropus spp*.), short-tailed shearwater (*Puffinus tenuirostris*), black-legged kittiwake (*Rissa tridactyla*), thick-billed murre (*Uria lomvia*), glaucous gull (*Larus hyperboreus*), and northern fulmar (*Fulmarus glacialis*). Because red-necked and red phalaropes often occur in mixed-species flocks and are difficult to distinguish at a distance, especially during molt, we combined observations of these 2 species with those of unidentified phalaropes and treated them collectively as phalaropes. These 8 focal taxa represented a variety of foraging methods (e.g., diving, surface feeding, shallow plunging) and prey preferences (e.g., planktivores, piscivores, omnivores), thereby providing an overview of the main functional ecological groups of the seabird community.

We considered 5 explanatory oceanographic variables to model the occurrence and abundance of the 8 focal taxa of seabirds. Hydrographic variables included temperature and salinity in the upper 10 m of the water-column, temperature and salinity in the lower 10 m of the water-column, and the density gradient from the surface to the bottom of the water column. Salinity and temperature are characteristics that define water masses in this region [19,62]. The density gradient is a characteristic of the water-column that we considered to be a proxy for foraging conditions. A strong density gradient indicates water column stratification that can enhance prey availability by concentrating prey at the pycnocline, whereas a weak density gradient can indicate a well-mixed water column that enhances prey availability at the surface [63–65]. These 5 variables were derived from measurements at fixed oceanographic stations throughout the study area. We assigned physical-oceanographic values to each transect based on the nearest station sampled to avoid artifacts inherent in using interpolated values. Values for each 30-km cell were calculated as the mean of values for each transect within a cell-season-year.

We also considered two time-related variables (year and season), and two geographic variables (latitude and distance from shore) calculated from the centroid of each grid cell in the models. We used distance to shore to account for the possible effect of proximity of terrestrial breeding habitat (coastal islands, cliffs, or tundra) that can influence foraging distributions of nesting marine birds. We did not include longitude because it was strongly correlated with distance from shore.

There were strong correlations (r > 0.6) among many combinations of the 5 water mass variables (temperature, salinity, and gradient). We therefore used principal component analysis (PCA) run on the scaled variables for variable reduction. The first component of the PCA analysis (PCA1) explained 69.7% of the variability in the 5 water mass variables. The PCA1 score increased with higher values of upper temperature, upper salinity, and bottom temperature, and decreased with higher values of density gradient and bottom salinity (Table 3).

**Table 3:** Factor loading output from principal component analysis of 5 oceanographic variables calculated over survey transects within hexagon shaped grid cells.

Variable	Comp.1	Comp.2	Comp.3	Comp.4	Comp.5
Temperature (upper 10 m)	0.464	–	0.882	–	–
Salinity (upper 10 m)	0.440	0.553	0.152	–	-0.684
Density gradient	-0.472	0.411	0.280	0.130	-0.717
Salinity (bottom 10 m)	-0.398	-0.622	0.224	0.627	0.106
Temperature (bottom 10 m)	0.458	0.369	-0.265	0.762	–
Proportion of variance explained	0.697	0.180	0.064	0.042	0.017

We used generalized additive models (GAM; [66]) to compare seabird counts to the two geographic factors (latitude and distance to shore) and the PCA1 score as an indicator of hydrographic conditions, hereafter, “hydrography”. We included year as a factor to account for variations in density among years. The count of each species of seabird within a hexagon was modeled with a negative binomial distribution and the natural log of transect area was included as an offset term to account for differing survey effort in different hexagons by year and season. GAM models were fit with the default smoother, a penalized thin plate regression spline [67].

We compared four different models containing the geographic variables (latitude and distance from shore) and hydrography: 1) a model with the geographic variables and hydrography varying by season; 2) a model with just the geographic variables varying by season; 3) a model with just hydrography varying by season, and 4) a model with no variables varying by seasons. We used the model with the lowest AIC score and highest model weight for inference [68].

## Results

### Oceanographic conditions

**Fig 3:**
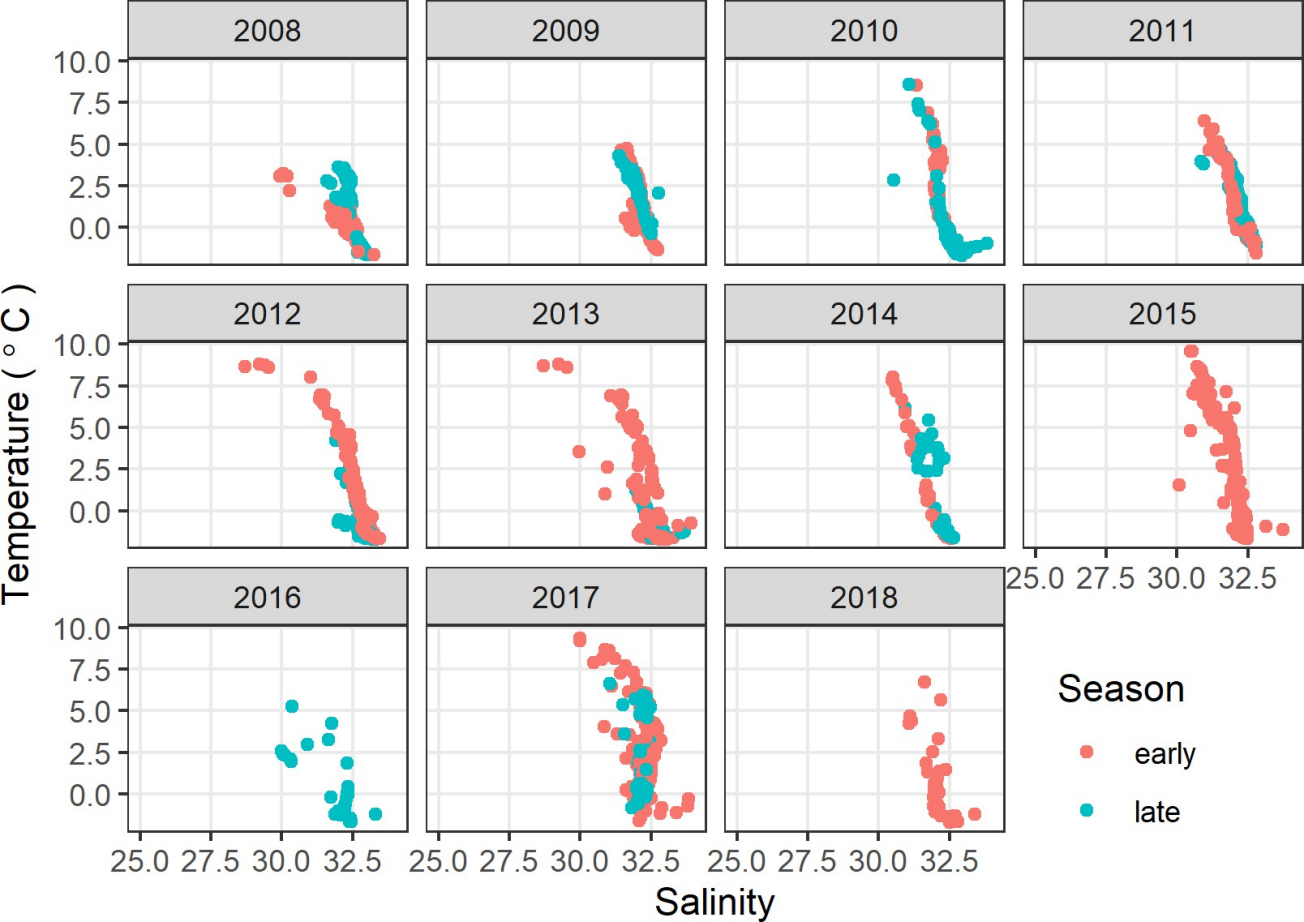
Temperature and salinity of water in bottom 10 m of water column, northeastern Chukchi Sea. Early summer was 13 Aug–6 September and late summer was 7 September–2 October.

**Fig 4:**
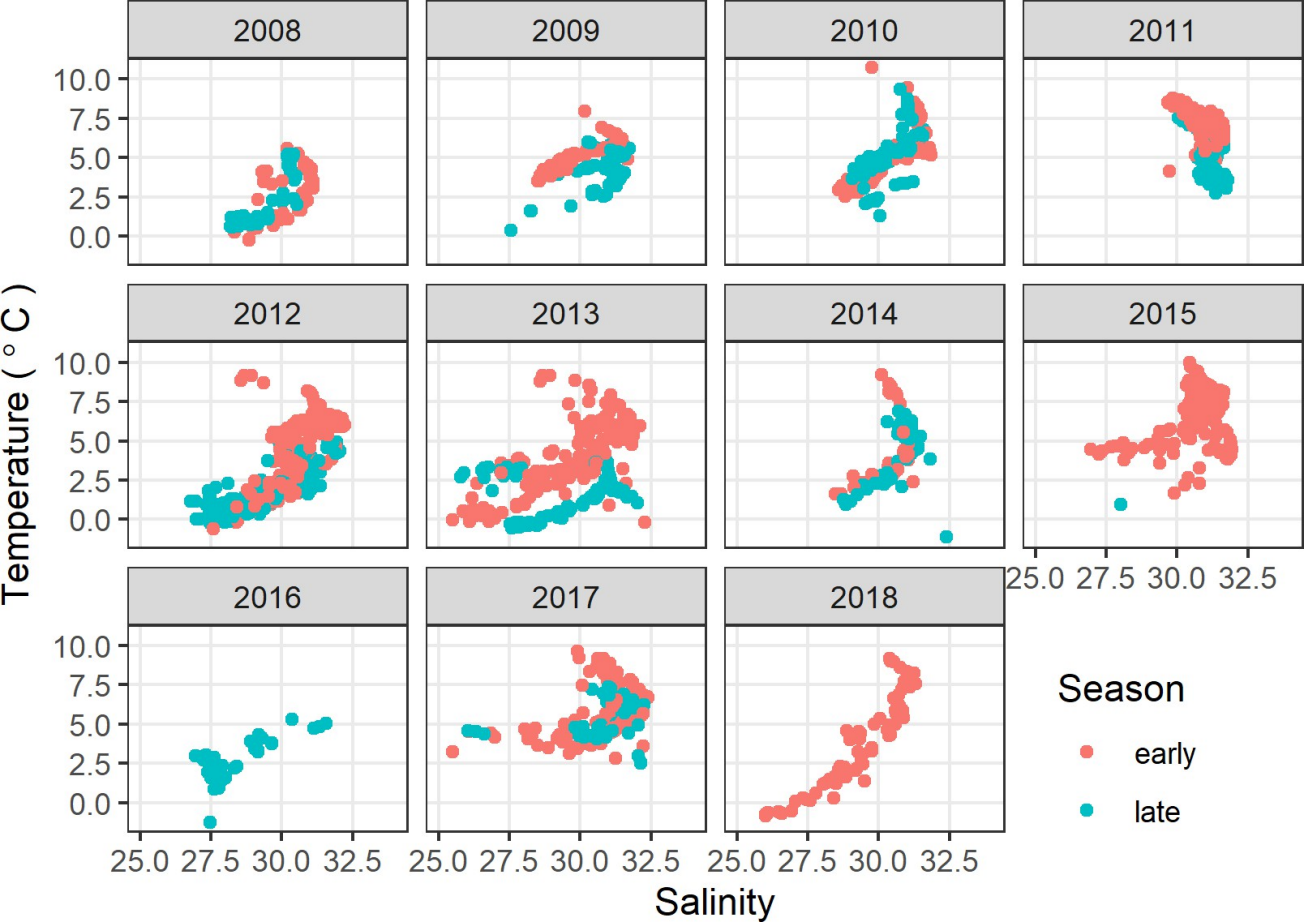
Temperature and salinity of water in upper 10 m of water column, northeastern Chukchi Sea. Early summer was 13 Aug–6 September and late summer was 7 September–2 October.

Denser near-bottom water (Fig 3) was generally cool (mean: 0.83°C, range: -1.72 to 9.82°C) and salty (mean: 32.4, range: 28.7 to 34.8) relative to the less dense surface water (Fig 4) that tended to be warmer (mean: 4.33°C, range: -1.12 to 10.06°C) and fresher (mean: 30.4, range: 25.5 to 32.4). In most years with sampling throughout the open-water period, surface water noticeably cooled from August to September (Fig 4), whereas in 2010 and 2017 temperatures in the upper 10 m of the water column remained relatively unchanged from the early to late season sampling.

### Seabird community

**Fig 5:**
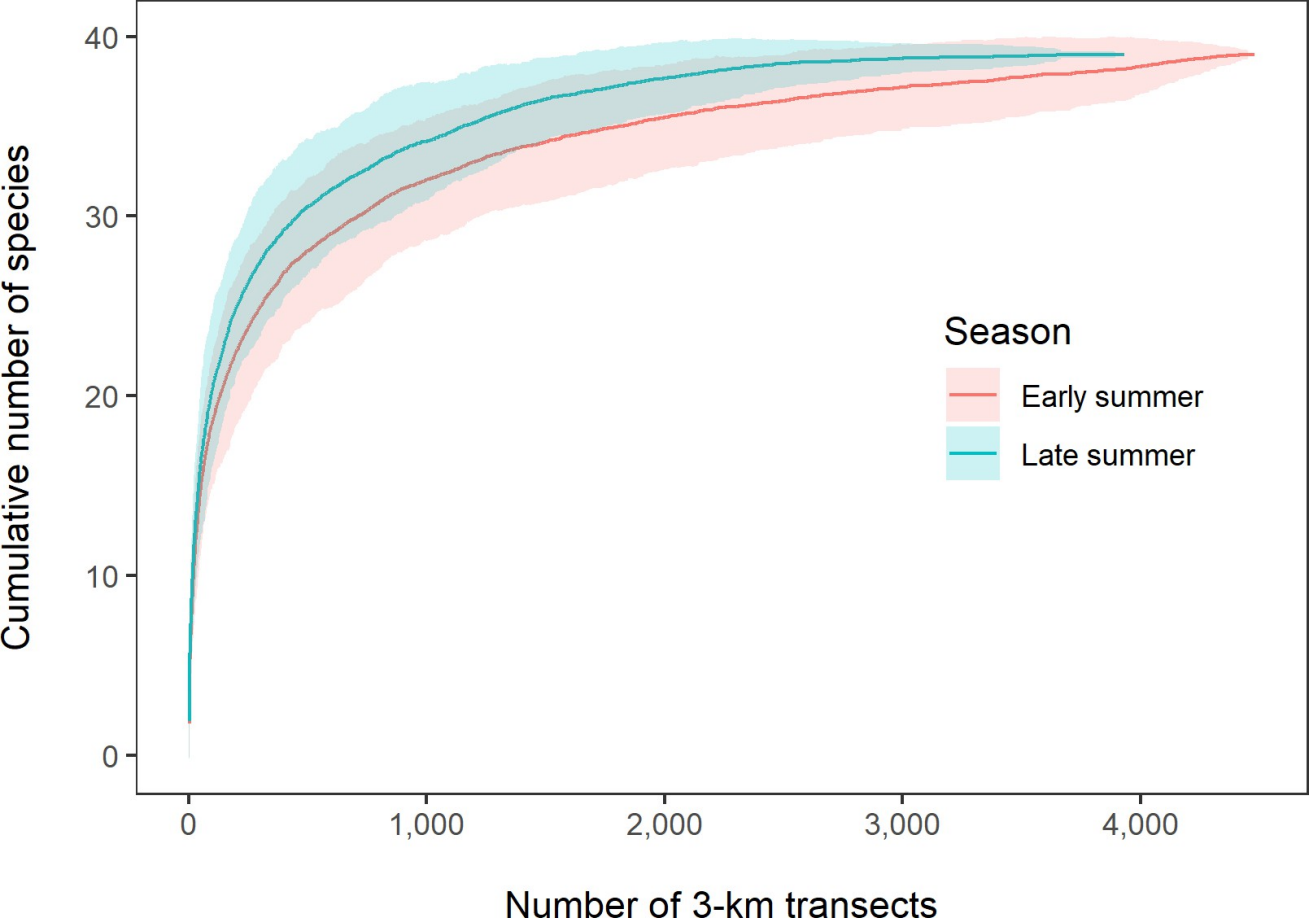
Seabird species rarefaction curves from surveys conducted in the northeastern Chukchi Sea in early and late summer, 2008–2018. Early summer (pink) was 13 August–6 September and late summer (aqua) was 7 September–2 October. Shading indicates 95% confidence intervals based on resampling transects without replacement.

We recorded a total of 90,985 individuals and identified 35 species of seabirds during these surveys. Of these, crested auklets were the most abundant (49% of total), followed by short-tailed shearwaters (31%) and least auklets (5%). Species richness was similar between seasons but slightly higher in late summer (Fig 5), with 33 and 35 species recorded in early and late summer, respectively. Ivory and Ross’s gulls were recorded only in late summer, all other species were recorded in both seasons.

**Fig 6:**
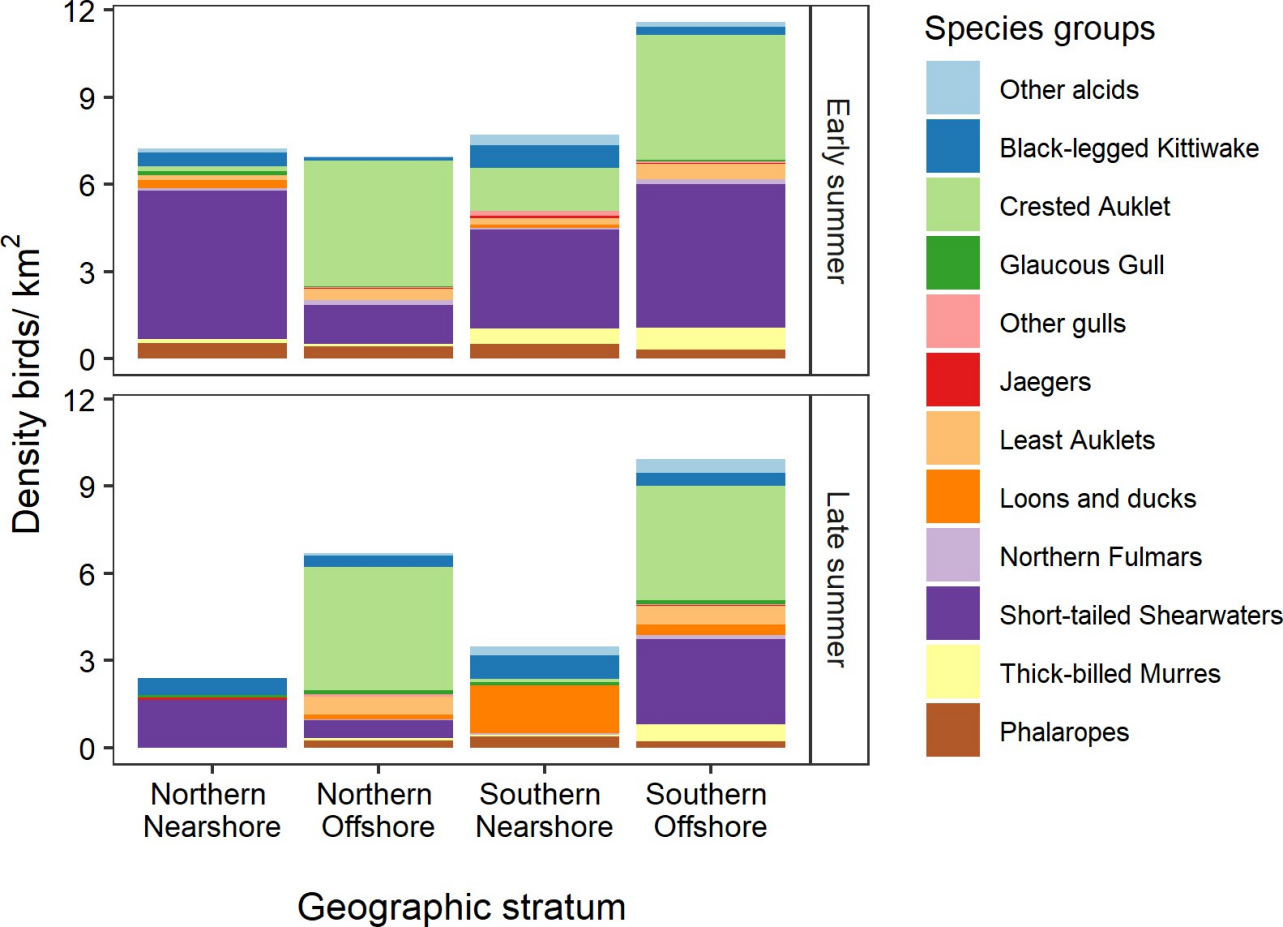
Species composition of seabird community in geographic strata of the northeastern Chukchi Sea, 2008–2018.

**Fig 7:**
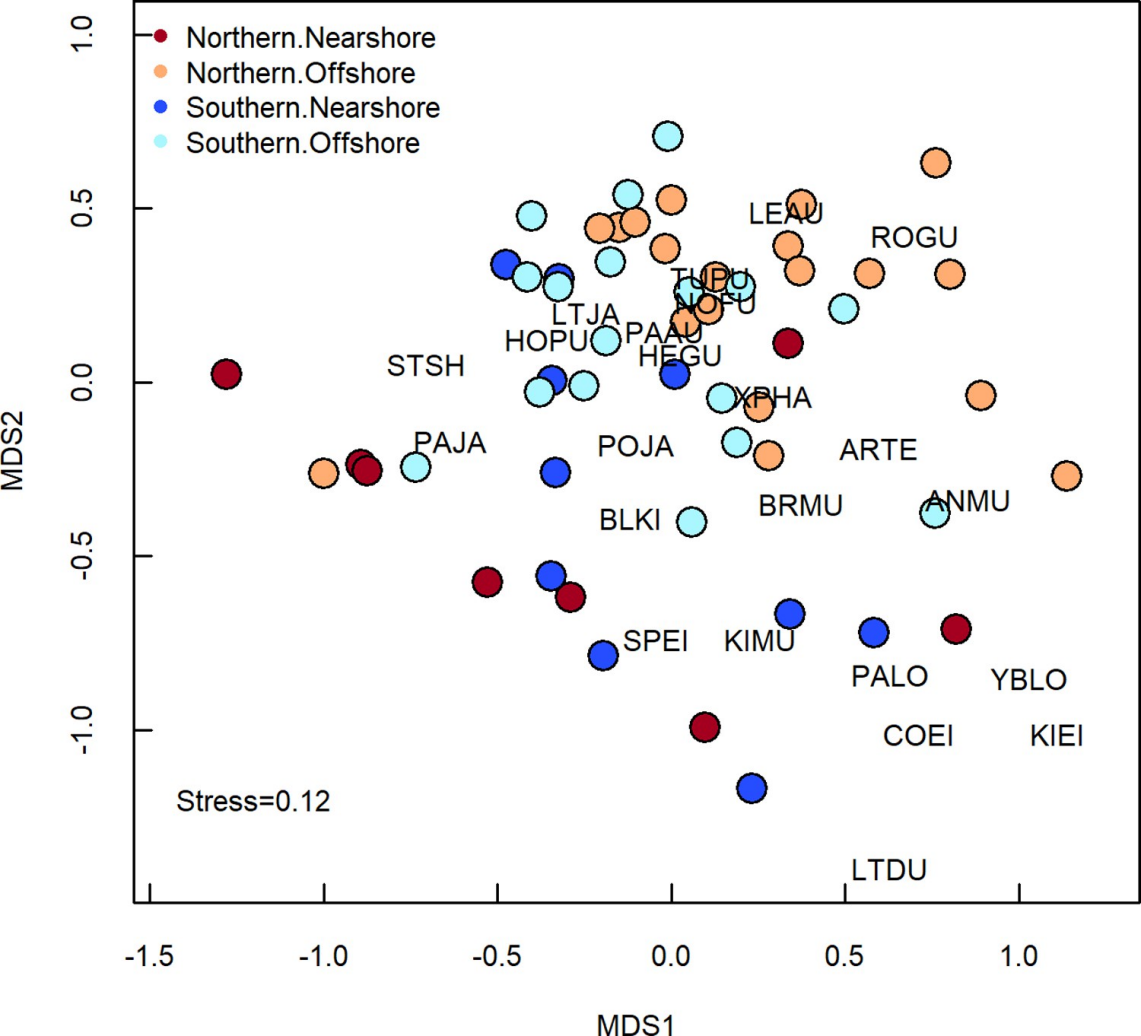
Non-metric multidimensional scaling (nMDS) of the seabird community in the northeastern Chukchi Sea, 2008–2018.

Species composition varied geographically (Table 4), shifting from a community that included short-tailed shearwaters, loons, and seaducks nearshore to one dominated by crested auklets offshore (Fig 6). The nMDS ordination showed a weak separation between the nearshore and offshore strata (Fig 7), with offshore areas having higher values along MDS1 and MDS2 than nearshore areas. Two season-year combinations were outliers from the predominant pattern. In early summer 2012, the species composition in the Northern Nearshore stratum included Least Auklets, phalaropes, and other alcids that are generally more abundant in the offshore areas. In late summer 2017, the first year of a 3-year heatwave, the species composition of the Northern Nearshore stratum clustered with the offshore samples because Short-tailed Shearwaters were more abundant and widespread than in other years, swamping out all other species in the Northern Nearshore, Northern Offshore, and Southern Offshore strata.

**Table 4:** PERMANOVA of species composition of the seabird community in the northeastern Chukchi Sea, 2008–2018.

Source	Degrees freedom	Mean squares	*F*	*R* ^2^	*P*
Season	1	0.467	2.083	0.035	0.046
Region	3	0.676	3.018	0.15	<0.001
Residuals	49	0.224		0.815	
Total	53			1.000	

Analysis was based on Bray-Curtis dissimilarities from log-transformed data. Each term was tested using 1,000 random permutations of the stratum-season-year samples.

### Seabird abundance and distribution

**Fig 8:**
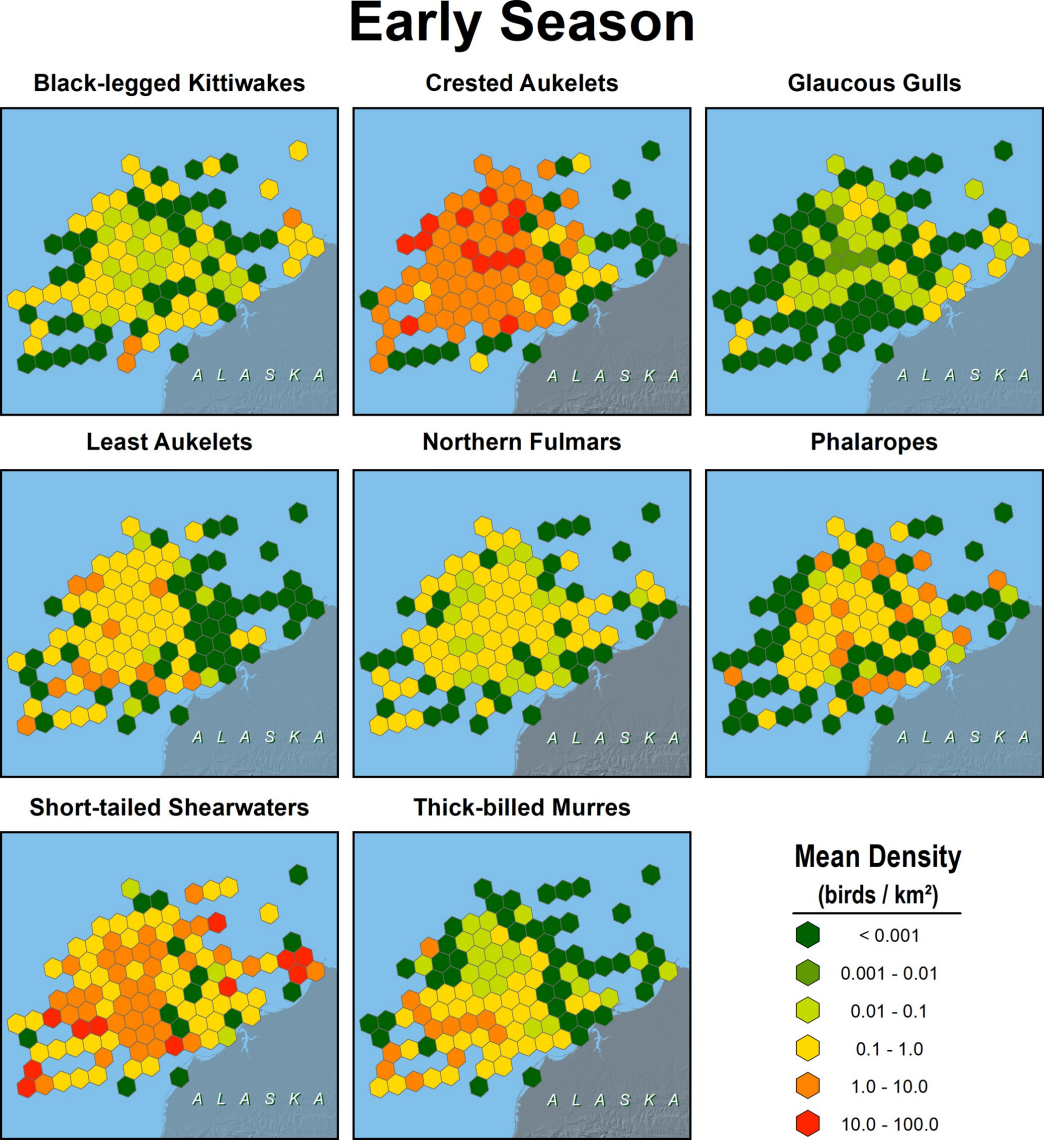
Distribution of 8 species of seabirds in the northeastern Chukchi Sea, early summer 2008–2018. Values in cells are means of transects from surveys conducted during 13 August–6 September.

**Fig 9:**
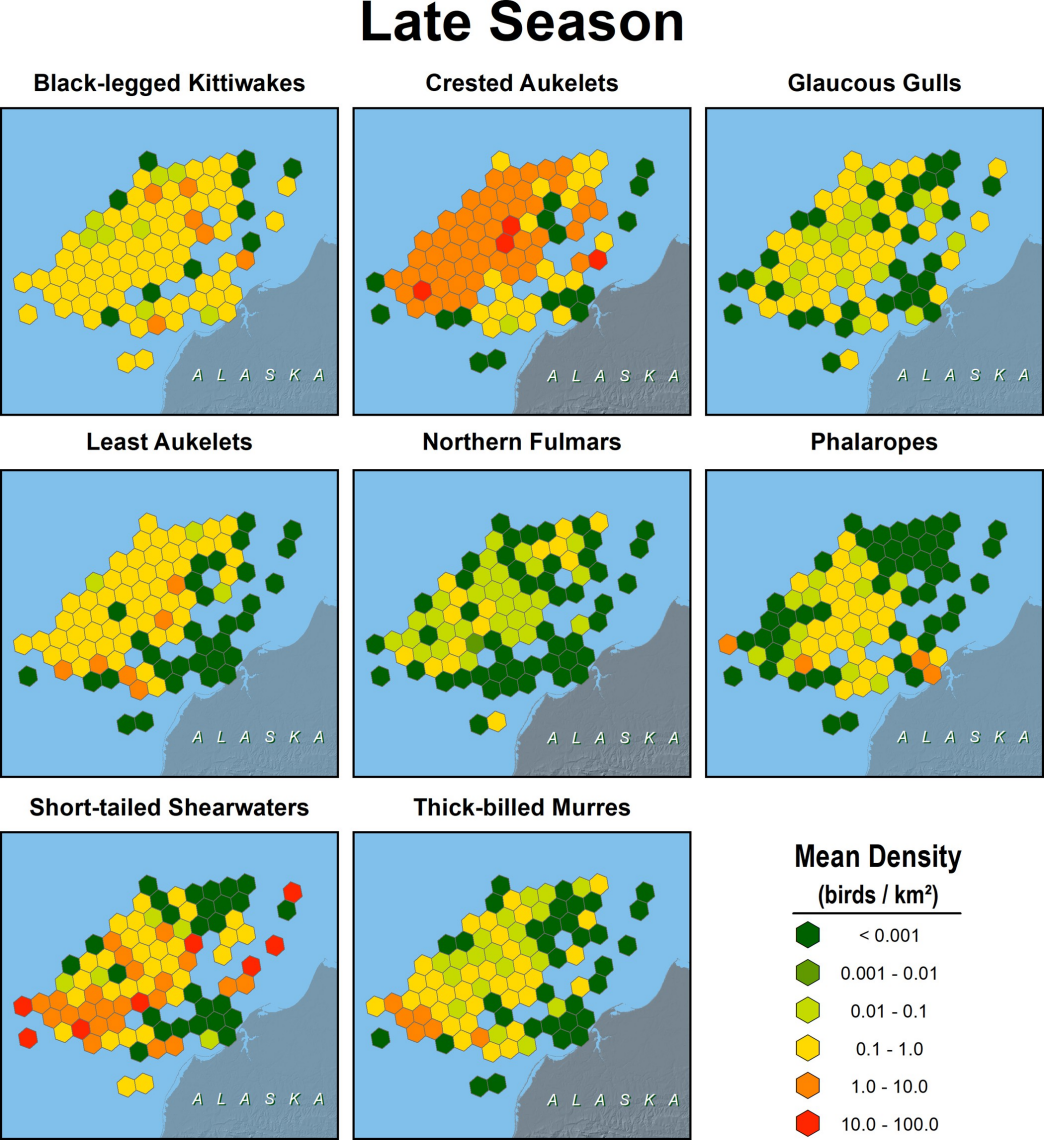
Distribution of 8 species of seabirds in the northeastern Chukchi Sea, late summer 2008–2018. Values in cells are means of transects from surveys conducted during 7 September–2 October.

Seabirds were more abundant offshore than nearshore, especially in early summer when short-tailed shearwaters were present in highest numbers. Least and crested auklets were more abundant offshore than nearshore in both seasons (Figs 8 and 9). Black-legged kittiwakes, short-tailed shearwaters, and phalaropes had areas of high abundance near Barrow Canyon in early summer. There was insufficient sampling in the nearshore area from Peard Bay north to Utqiaġvik to quantify patterns in seabird density near Barrow Canyon in late summer.

**Fig 10:**
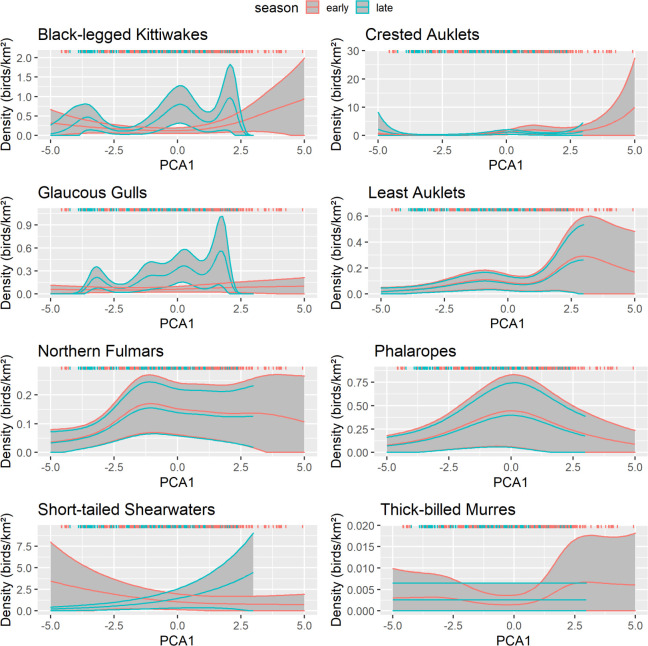
Effect of hydrography on seabird density, northeastern Chukchi Sea, 2008–2018. Response curves are from the best-fitting generalized additive model. Solid lines represent the smooth function and 95% confidence limits for early summer (pink) and late summer (aqua). Gray shading indicates the 95% confidence intervals. Colored ticks indicate the distribution of observations.

For 4 of 8 species, the best predictive model for abundance included the geographic variables (latitude, distance to shore), hydrography, and interactions with season. The model with hydrography and interactions between season and the geographic variables was the best model for 3 species, and the model with geographic variables and an interaction between season and hydrography was the best model for thick-billed murre (Table 5). There was model uncertainty for phalaropes and glaucous gulls, with two models nearly equal in performance (Table 5), although parameter estimates did not support a strong seasonal difference in the effect of hydrography (Fig 10).

**Table 5:** Generalized additive models that best explained variation in abundance and distribution of seabirds in the northeastern Chukchi Sea, 2008–2018 (n = 256 hexagonal cells, 30-km from vertex to vertex).

Model	AIC	ΔAIC	ω_*i*_
*Phalaropes*			
Hydrography; DistShore (seasons), Latitude (seasons)	2248.0	0.0	0.53
Hydrography (seasons); DistShore (seasons), Latitude (seasons)	2248.9	0.9	0.34
Hydrography, DistShore, Latitude	2251.4	3.4	0.10
Hydrography (seasons); DistShore, Latitude	2254.0	6.0	0.03
*Thick-billed Murres*			
Hydrography (seasons); DistShore, Latitude	1902.7	0.0	0.87
Hydrography (seasons); DistShore (seasons), Latitude (seasons)	1906.5	3.8	0.13
Hydrography, DistShore, Latitude	1914.6	11.9	0.00
Hydrography; DistShore (seasons), Latitude (seasons)	1918.5	15.8	0.00
*Least Auklets*			
Hydrography; DistShore (seasons), Latitude (seasons)	2523.2	0.0	0.94
Hydrography (seasons); DistShore, Latitude	2530.1	6.9	0.03
Hydrography, DistShore, Latitude	2531.4	8.1	0.02
Hydrography (seasons); DistShore (seasons), Latitude (seasons)	2532.4	9.2	0.01
*Crested Auklets*			
Hydrography (seasons); DistShore (seasons), Latitude (seasons)	4912.3	0.0	1.00
Hydrography; DistShore (seasons), Latitude (seasons)	4928.2	15.9	0.00
Hydrography (seasons); DistShore, Latitude	4930.3	18.0	0.00
Hydrography, DistShore, Latitude	4943.5	31.2	0.00
*Black-legged Kittiwakes*			
Hydrography (seasons); DistShore, Latitude	2485.6	0.0	1.00
Hydrography (seasons); DistShore (seasons), Latitude (seasons)	2504.3	18.7	0.00
Hydrography; DistShore (seasons), Latitude (seasons)	2562.6	77.1	0.00
Hydrography, DistShore, Latitude	2562.9	77.4	0.00
*Glaucous Gulls*			
Hydrography (seasons); DistShore (seasons), Latitude (seasons)	1358.1	0.0	0.53
Hydrography (seasons); DistShore, Latitude	1358.4	0.3	0.47
Hydrography; DistShore (seasons), Latitude (seasons)	1433.9	75.8	0.00
Hydrography, DistShore, Latitude	1443.0	84.9	0.00
*Northern Fulmars*			
Hydrography; DistShore (seasons), Latitude (seasons)	1777.5	0.0	0.69
Hydrography, DistShore, Latitude	1779.4	1.8	0.28
Hydrography (seasons); DistShore (seasons), Latitude (seasons)	1784.6	7.1	0.02
Hydrography (seasons); DistShore, Latitude	1786.5	9.0	0.01
*Short-tailed Shearwaters*			
Hydrography (seasons); DistShore (seasons), Latitude (seasons)	3852.9	0.0	1.00
Hydrography; DistShore (seasons), Latitude (seasons)	3872.0	19.1	0.00
Hydrography (seasons); DistShore, Latitude	3875.5	22.6	0.00
Hydrography, DistShore, Latitude	3888.2	35.3	0.00

Values are the Akaike’s Information Criterion score (AIC), difference in AIC score (ΔAIC) from the the model with the best fit, and Akaike weights (ω_*i*_). DistShore is the distance to shore from the centroid of each cell in the sampling grid.

Hydrography was a significant predictor of seabird distribution in most cases, with the exceptions of glaucous gull in early season (p = 0.085) and thick-billed murre in late season (p = 0.062; Table 6). After accounting for latitude and distance to shore, black-legged kittiwakes, crested auklets, least auklets, northern fulmars, and thick-billed murres were all positively associated with areas that had warmer, saltier water in the upper layer and weaker density gradients in early summer (Fig 10). These conditions were typical of BSW in the Central Channel and other offshore areas. For auklets, northern fulmars, and phalaropes, the effect of hydrography was consistent among seasons. In contrast, densities of short-tailed shearwaters in early summer were positively associated with water that was cooler, fresher, and more stratified, suggesting an association with ACW. In late summer, short-tailed shearwaters were positively associated with waters that warmer and saltier in the upper layer (Fig 10), which was indicative of BSW.

**Table 6:** Variables that best described the variation in distribution and abundance of 8 species of seabirds in the northeastern Chukchi Sea, 2008–2018. P-values indicate statistical significance from generalized additive models (GAM). Dist. Shore is the distance to shore from the centroid of each cell in the sampling grid.

Variable	Phalaropes	Thick-billed Murres	Least Auklets	Crested Auklets	Black-legged Kittiwakes	Glaucous Gulls	Northern Fulmars	Short-tailed Shearwaters
Hydrography	**<0.001**		**<0.001**				**0.001**	
Hydrography: Early summer		**<0.001**		**<0.001**	**<0.001**	0.085		**0.021**
Hydrography: Late summer		0.692		**<0.001**	**<0.001**	**<0.001**		**<0.001**
Dist. Shore		**0.001**			0.403			
Dist. Shore: Early	0.578		**<0.001**	**<0.001**		**<0.001**	0.067	**<0.001**
Dist. Shore: Late	0.238		**<0.001**	**<0.001**		**0.050**	**0.039**	**0.009**
Latitude		**<0.001**			0.271			
Latitude: Early	**0.045**		**0.034**	**<0.001**		**0.003**	0.061	**<0.001**
Latitude: Late	**0.035**		**<0.001**	**<0.001**		**0.050**	0.654	0.634
Deviance Explained	**19.10%**	**54.18%**	**47.77%**	**35.33%**	23.48%	28.68%	21.60%	30.23%

**Fig 11:**
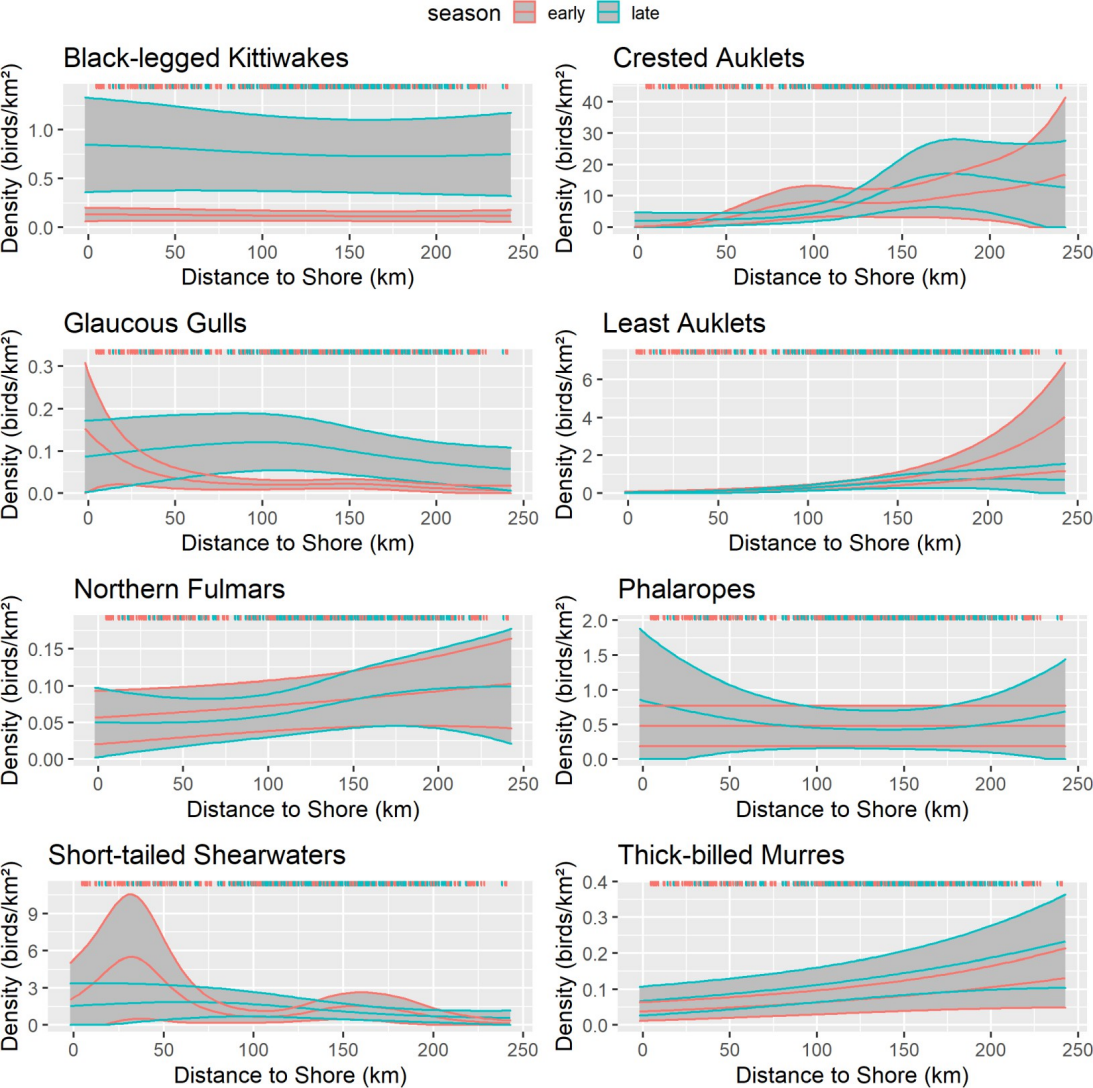
Effect of distance to shore on seabird density, northeastern Chukchi Sea, 2008–2018. Response curves are from the best-fitting generalized additive model. Solid lines represent the smooth function and 95% confidence limits for early summer(pink) and late summer(aqua). Gray shading indicates the 95% confidence intervals. Colored ticks indicate the distribution of observations.

The distance to shore variable was significant for all species except black-legged kittiwakes (p = 0.201) and northern fulmars during early summer (p = 0.067), and phalaropes during both the early summer (p = 0.578) and late summer (p = 0.238; Table 6). Crested and least auklets and thick-billed murres were more abundant farther offshore in both seasons whereas glaucous gulls and short-tailed shearwaters were more abundant nearshore in early summer and distributed throughout the study area in late summer (Fig 11).

**Fig 12:**
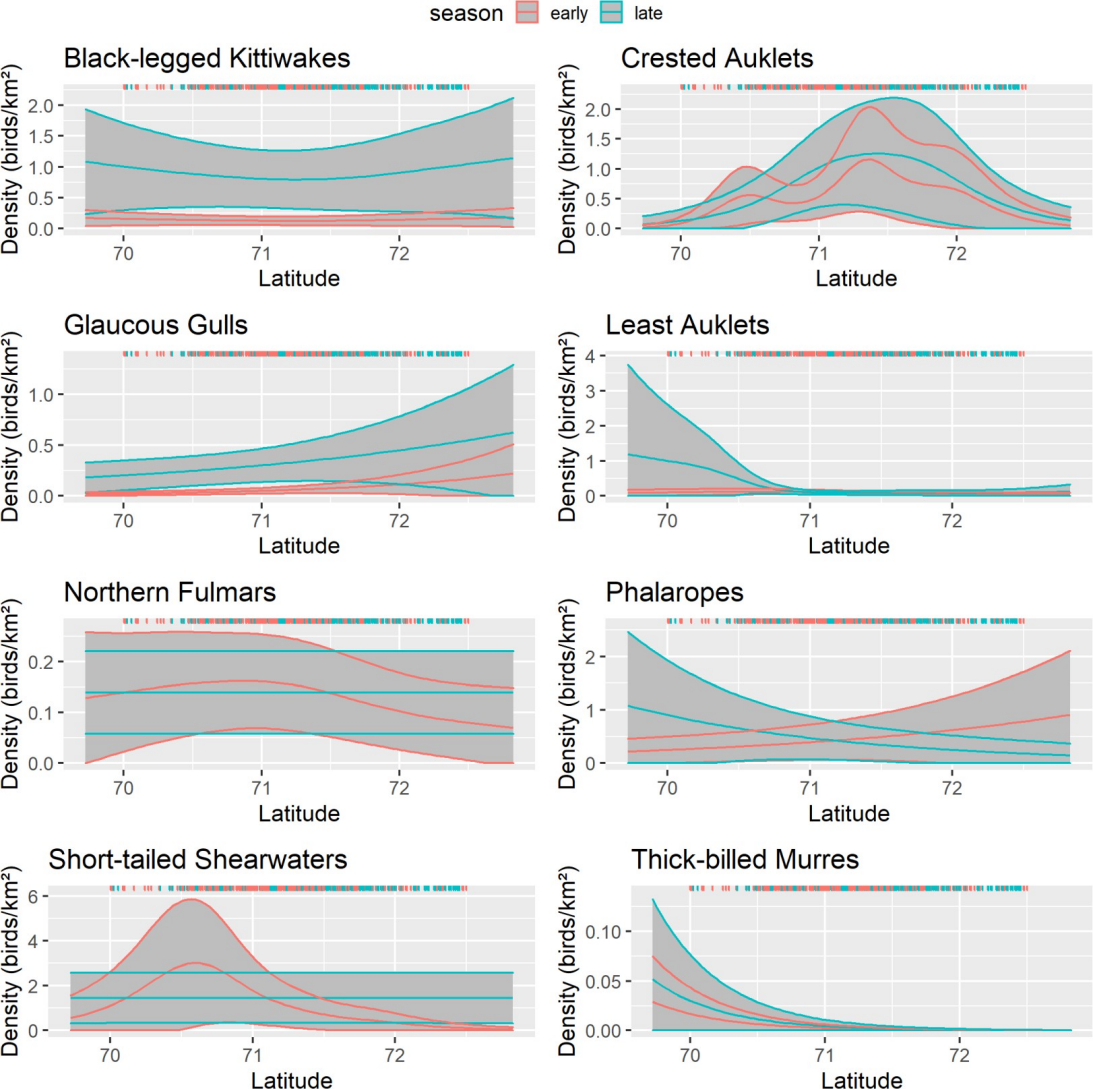
Effect of latitude on seabird density, northeastern Chukchi Sea, 2008–2018. Response curves are from the best-fitting generalized additive model. Solid lines represent the smooth function and 95% confidence limits for early summer(pink) and late summer(aqua). Gray shading indicates the 95% confidence intervals. Colored ticks indicate the distribution of observations.

The latitude variable was significant for all species except black-legged kittiwakes (p = 0.136), northern fulmars during the early season (p = 0.061) and late season (p = 0.654), and short-tailed shearwaters during the late season (p = 0.634; Table 6). Least Auklets, phalaropes, and thick-billed murres were more abundant south of 71°N in late summer whereas black-legged kittiwakes, crested auklets, glaucous gulls, and northern fulmars had similar or higher abundance north of 71°N in late summer as well as in early summer (Fig 12). Short-tailed Shearwaters were generally more abundant south of 71°N in both seasons (Figs 8 and 9). The widespread distribution of Short-tailed Shearwaters in late summer 2017 may have influenced the estimate of the effect of latitude in the models (Fig 12).

## Discussion

We show that the distribution of seabirds throughout the northeastern Chukchi Sea reflects the heterogeneity of oceanic habitats over the shallow shelf. Auklets, murres, and northern fulmars generally were more abundant in offshore areas of the Chukchi Sea dominated by moderate-salinity Bering Sea Water than nearshore in low-salinity Alaska Coastal Water. The seabird community within 50 km of the coast had low densities compared to areas farther offshore. Although auklets and other alcids were found in these nearshore waters, the community was composed primarily of short-tailed shearwaters and also included diving piscivores such as loons and benthic feeders such as eiders and long-tailed ducks.

With a maximum depth of only ~ 100m and most of the shelf < 50m deep, variations in bathymetry of only a few meters help steer water masses of varying temperature and salinity, and their associated prey species [24]. High salinity surface currents flowing northward through the Central Channel from the Bering Sea carry copepods and euphausiids to the Chukchi Sea [15,34]. Auklets (*Aethia* spp) and short-tailed shearwaters that forage in the Bering Sea from May through July were found in the Central Channel stream in August and September, presumably following their prey [11,12,69].

As with other studies of seabird distribution [70], GAMs were effective at revealing the factors that caused observed variance in seabird abundance. The GAMs revealed that most seabird-habitat relationships were species-specific, non-linear, and in some cases, varied by season. In general, auklets and murres, species that feed almost exclusively by diving, had distributions that were well-predicted by latitude and distance to shore. Thick-billed murres and least auklets were more abundant south of 71°N whereas crested auklets were distributed primarily 71–72°N throughout the summer. In contrast, short-tailed shearwaters were more abundant south of 71°N in early summer and at all latitudes in late summer. Although short-tailed shearwaters are capable divers [71], they are also stronger fliers than are alcids.

Surface-feeding species like gulls, fulmars, and phalaropes had wider distributions than birds that feed by diving. Glaucous gulls nest on the Arctic Coastal Plain and may have been more abundant nearshore in August because they were still tending to chicks. By September, most glaucous gull young have left the nest and are independent [72], allowing both adults and young to disperse widely. The distributions of black-legged kittiwakes and phalaropes were not influenced by distance to shore.

Including information about hydrography improved the fit of the models describing seabird density, despite the challenges of quantifying oceanographic conditions at scales that match the decisions made by foraging seabirds. The relationship to hydrography was strongest and most consistent between seasons for northern fulmars, phalaropes, and least auklets. Thick-billed murres, however, were associated with BSW in early summer but showed no relationship to hydrography in late summer. The relationship of surface-feeding species to hydrography was more challenging to characterize, partly because they had low abundance overall and perhaps because they may make decisions about foraging at spatial and temporal scales that are shorter than those at which hydrography was sampled in this study [50,73,74].

### Influence of foraging conditions

We assumed that foraging conditions were the most important factor in determining the distribution of seabirds. During the early summer (which was primarily August in this study), we observed consistent associations of planktivorous seabirds with offshore waters that are typically saltier than waters found along the coast. In late summer (primarily September in this study), we observed southward movements in species such as phalaropes and thick-billed murres, while auklets and gulls remained widespread throughout the study area. This southward movement was consistent with a southward shift during fall that was described for seabirds using the Chukchi Sea in 2007–2012 [12]. The timing of departure from the northeastern Chukchi Sea precedes the formation of ice by several weeks, suggesting that the availability of preferred prey for these southbound species changes sooner than it does for auklets.

Crested auklets numerically dominated the seabird community in the northeastern Chukchi Sea throughout the open-water season in most years, even though their nearest nesting areas were at least 550 km to the south. Our study area closely overlapped the ‘crested auklet-dominated’ community identified within a larger study area encompassing the northern Bering and Chukchi seas [69]. This was one of five communities defined for the Pacific Arctic overall, and one of the most spatially well defined, indicating specific habitat preferences, or with prey associated with that habitat. Crested auklets are widespread across the Chukchi shelf and least abundant nearshore. They are remarkably consistent in their occupation of Hanna Shoal and remain in the area until ice starts to form in October [12,69]. Observations during surveys suggest that crested auklets are flightless and likely undergoing molt during August and early September, which limits their mobility and makes it even more important that prey be reliably accessible. Other diving species that rely heavily on planktonic prey, such as short-tailed shearwaters and thick-billed murres, are also common offshore but do not aggregate as far north as do crested auklets. What is it about Hanna Shoal that attracts such high numbers of crested auklets?

The zooplankton community around and south of Hanna Shoal is dominated by *Calanus glacialis* and *Pseudocalanus* spp. [34,75,76], prey that are essential to crested auklets [77,78]. Hanna Shoal is encircled by clockwise circulation that brings BSW northward along the western flank and then east towards Barrow Canyon [22,79,80]. To the east, water from the Shoal mixes with northward flowing coastal currents [75]. These general patterns of circulation can vary in their persistence and strength among years, leading to variable mixing of water masses and their entrained zooplankton [34,75]. The combination of shallow bathymetry, weak surface flow, and reliable aggregations of zooplankton advected from the Bering Sea make the eastern Chukchi Sea ideal habitat for non-breeding and post-breeding crested auklets.

Together with crested auklets, short-tailed shearwaters drive community structure in the northeastern Chukchi Sea. In contrast to crested auklets, short-tailed shearwaters were less consistent in distribution and abundance among years. This greater inter-annual variance compared to location was also evident at a larger geographic scale study that included all DBO sites [69]. In most years, short-tailed shearwaters are strongly associated with nearshore waters south of 71°N. The exceptions were occasional years (2009, 2017) when shearwaters were extremely abundant and dispersed widely, occupying more northerly and/or offshore regions. In the Chukchi Sea, shearwaters appear to forage primarily on euphausiids [81], although they also consume large zooplankton, invertebrates and small fish [82]. Seabird surveys of the northern Bering and Chukchi seas showed a trend of northward movement of short-tailed shearwaters beginning around 2013, with peak numbers in 2015 [69], thus a pattern of greater occupation of the Chukchi Sea by shearwaters began prior to the large influx we observed in 2017. However, 2017 was the first of a 3-year period with exceptionally warm ocean waters in the northern Bering-Chukchi large marine area [5,55]. During this period, seabird die offs occurred, breeding seabirds failed, and some species showed declines in abundance at sea [83,84]. Concurrently, small copepods predominated in place of large-bodied copepods, and they occurred farther north in the Chukchi Sea. In a study focused on the Barrow Canyon area of the northern Chukchi Sea, krill abundance showed a positive correlation between late spring ice melt and ice extent, with those conditions occurring in 2006, 2009, 2012–2014, and the opposite occurring other years through 2015 [85]. Although 2017 was outside the time periods examined, these results suggest that years of shearwater irruptions in the Chukchi Sea (2009, 2013) may coincide with high krill abundance driven by spring ice conditions.

### Geographic patterns

Effective marine conservation relies on the predictability of locating resources that require protection. One of the essential assumptions of the DBO is that the sites selected for monitoring are in areas of high biomass, high species biodiversity, representative of the Pacific Arctic ecosystem, and will remain so over time [40,86]. This study focused on DBO sites 4 and 5, where hotspots of seabird aggregation have been identified in nearshore waters near the village of Wainwright, in an offshore area on the southern flank of Hanna Shoal, and at the mouth of Barrow Canyon [12,69]. These hotspots were also apparent in our analysis in early summer. In late summer, however, we did not include data from near Barrow Canyon because none of the transects in that area had oceanographic data available from within 2 days and 20 km of when the birds were recorded. Our study emphasizes the importance of collecting data on seabird occurrence concurrently with oceanographic data on water column properties, currents, and perhaps most importantly, thermohaline fronts that affect prey availability. Doing so will improve our ability to predict possible future shifts in the distribution and abundance of seabirds as the Artic warms. Our results can inform efforts to develop ecosystem models that incorporate oceanographic conditions, nutrients, prey species, and top predators to predict ongoing consequences of climate change [87].

## Supporting information

S1 Dataset Observations of seabirds and measurements of physical oceanography in the northeastern Chukchi Sea. (CSV)
